# Cyclopropylamine‐Derived Distonic Iminium Radicals: Photoinduced Strategies in Chemo‐ and Stereo‐Selective Synthesis

**DOI:** 10.1002/chem.71383

**Published:** 2026-07-12

**Authors:** Maria Chiara Cabua, Alberto Luridiana, Francesco Secci, Emanuele Cocco

**Affiliations:** ^1^ Department of Chemical and Geological Sciences Università degli Studi di Cagliari Complesso Universitario di Monserrato Monserrato Cagliari Italy

**Keywords:** [3 + 2] Cycloaddition, cyclopropylamin, photocatalysis, photochemistry, β‐iminium radical cation

## Abstract

Cyclopropylamines have emerged as privileged substrates in photocatalysis owing to their propensity to undergo single‑electron oxidation followed by strain‑release β‐scission, generating highly reactive distonic iminium radicals. These intermediates underpin a broad repertoire of light‐driven transformations, including ring‑opening functionalization, formal [3 + 2] cycloadditions, radical cascade processes, and asymmetric bond‐forming reactions. This Review highlights recent advances in the photochemical activation of cyclopropylamines, emphasizing mechanistic insights, catalyst design, and the synthetic potential of these reactions for accessing complex carbocycles, heterocycles, and medicinally relevant scaffolds. This review is organized to first summarize the preparation of cyclopropylamine derivatives, then to discuss their photoinduced rearrangements and ring‑opening pathways, followed by an overview of [3 + 2] cycloadditions, enantioselective strategies, and applications for constructing unconventional building blocks. Together, these developments underscore the growing impact of cyclopropylamine‐derived radicals as versatile tools in modern synthetic methodology.

## Graphical abstract

Cyclopropylamines have emerged as powerful photocatalytic building blocks. Upon light‐induced single‐electron oxidation, they undergo strain‐release ring opening to generate distonic iminium radicals that enable diverse transformations, including ring‐opening functionalization, formal [3 + 2] cycloadditions, radical cascades, and asymmetric bond‐forming reactions.

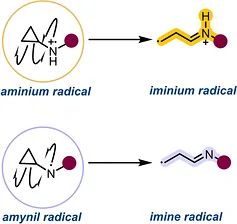

## 1 Introduction

The chemistry of cyclopropane derivatives has been extensively explored over the past decades [1]. Early developments centered on thermal strategies employing transition metals [2, 3, 4], oxidation reactions [5], and electrophilic or nucleophilic ring‐opening and ring‐expansion processes [6, 7, 8, 9, 10]. The prominence of strained carbocycles in biologically active molecules [11], natural products [12, 13], agrochemicals [14], and complex synthetic targets [15, 16] has further stimulated efforts toward their synthesis and functionalization. More recently, advances in organocatalysis [17, 18] and photocatalysis [19] have transformed the field, offering sustainable and versatile approaches for molecular construction. In particular, photoredox catalysis has unveiled unprecedented reactivity of cyclopropane derivatives, greatly expanding the synthetic opportunities available from these highly strained frameworks [20, 21, 22, 23]. Photocatalytic ring‐expansion reactions of cyclopropyl scaffolds under visible‐light irradiation have emerged as powerful routes to heterocycles [24], while tandem ring‐opening/intramolecular trapping processes enable the synthesis of enantioenriched polycyclic cyclopentane derivatives [25]. In this context, vinyl cyclopropanes [1, 26, 27] undergo photoinduced C─C bond cleavage upon UV irradiation to generate allyl‐stabilized biradicals (I) [28]. The reactivity of these intermediates is highly dependent on the reaction conditions, providing access to a broad range of molecular architectures (Figure 1a).

**FIGURE 1:**
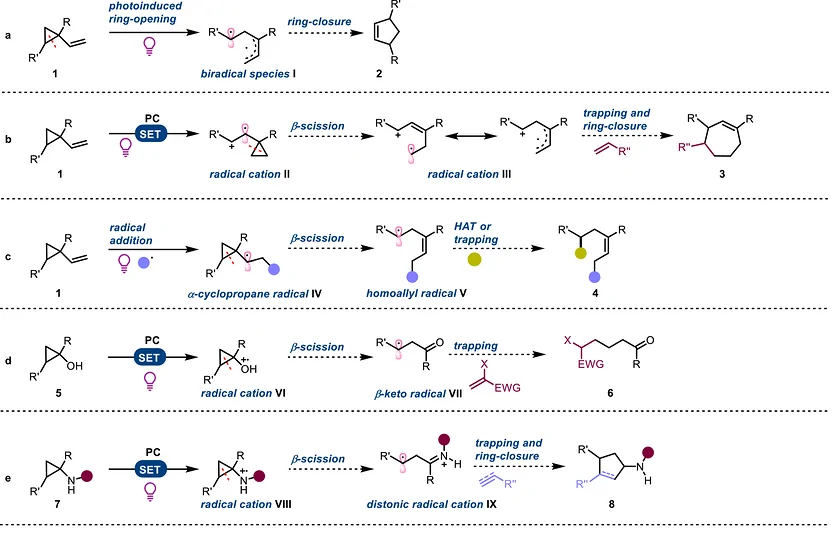
Photoinduced generation of radical species from cyclopropane derivatives.

More recently, photoredox catalysis has significantly expanded the synthetic potential of vinyl cyclopropanes. Trapping of the resulting radical intermediates (**II** and **III**) with olefins furnishes carbocyclic frameworks of varying ring size [29, 30] (Figure 1b), whereas acceptor‐substituted derivatives (**IV**) react with electrophilic or acyl radicals [14, 15, 16, 17, 18, 19, 20, 21, 22, 23, 24, 25, 26, 27, 28, 29, 30, 31] or undergo photopolymerization processes [32] to afford linear products 4 (Figure 1c).

Complementary reactivity is observed for electron‐rich cyclopropanes. Photoredox oxidation of cyclopropanols [33, 34]. 5 generates β‐ketoalkyl radicals (**VII**), which readily add to substituted alkenes (Figure 1d) [35], providing *γ*‐substituted ketones [36, 37] or, through radical coupling pathways, polysubstituted cyclopentyl aldehydes [38]. Similarly, cyclopropylamines **7** have emerged as versatile building blocks owing to their ability to stabilize radical cation intermediates (Figure 1e). Upon photoinduced, oxidative, or electrochemical activation, these substrates undergo single‐electron oxidation (**VIII**), triggering β‐scission to generate reactive iminium radicals **IX** [39, 40, 41]. These intermediates readily engage with alkenes, alkynes, and related *π*‐systems, enabling the formation of structurally complex products. Notably, photochemical ring opening of 2‐phenylcyclopropylamines yields intermediates prone to recombination, often leading to stereochemical scrambling [42].

As cyclopropylamines lack intrinsic visible‐light absorption, efficient activation relies on photocatalysts with appropriate redox potentials and quenching kinetics to promote electron transfer. Transition‐metal‐based photocatalysts, such as Ru(II) and Ir(III) complexes, and organic photocatalysts, including 4CzIPN, 4DPAIPN, Eosin Y, and DPZ, have been widely employed for this purpose [43, 44, 45]. Additionally, UV‐driven intramolecular cyclopropylamine transformations have been demonstrated using stoichiometric sensitizers, such as DCB [46, 47] in early reports and at the onset of the photoredox catalysis boom [48, 49].

Upon absorbing visible light, photocatalysts are excited to singlet states (*S*_1_), which can undergo intersystem crossing (ISC) to long‐lived triplet states (*T*_1_). In their triplet‐excited states, these radical‐like photocatalysts can participate in electron transfer with amines, leading to either reductive quenching (electron donation) or oxidative quenching (electron acceptance) [49, 50]. This process results in the formation of radical cation intermediates, which are capable of undergoing further reactivity. Early studies, particularly those conducted by Williams and coworkers, demonstrated the formation of cyclopropylamine radical cations through electron spin resonance (ESR) experiments [51]. These radical cations were shown to weaken C─C bonds, facilitating strain‐driven ring‐opening reactions. Subsequent works by Mariano and others, applied these principles to explain the role of radical cation generation in substrate inhibition within enzymatic systems [52]. Recent advances in photocatalysis have further refined these concepts, enabling greener and milder synthetic routes [53].

Light‐driven reactions not only circumvent the need for stoichiometric reagents and high temperatures but also broaden the scope of accessible products for industrial applications [54]. Additionally, protocols for photocatalyst regeneration have been developed to ensure catalytic efficiency. This is often achieved through reoxidation by molecular oxygen or halogen anions generated in situ [55, 56].

This review aims to summarize recent progress in the light‐driven transformations of cyclopropylamines, focusing on their unique reactivity and role as intermediates in complex reaction pathways. These pathways enable access to complex molecular architectures and the production of highly valuable building blocks from both synthetic and application‐oriented perspectives. To this end, we have structured this work by first considering the synthetic strategies used to access various cyclopropylamine derivatives. We then proceed to describe the different photoinduced approaches reported in the literature to date.

### 1.1 Synthesis of Cyclopropylamine Derivatives

Before reviewing the various synthetic procedures described in this work, it is essential to consider the most commonly used methodologies for the preparation of cyclopropylamine derivatives. Despite the growing availability of these derivatives in the market, transition metal‐catalyzed cross‐coupling reactions (Cu, Pd, and Ni) stand out as the preferred methods, especially for introducing aryl moieties on the nitrogen of cyclopropylamines (Scheme 1a,g) [57, 58, 59, 60, 61, 62, 63]. Another strategy for synthesizing these compounds involves SNAr reactions [64, 65, 66] on fluorobenzenes (Scheme 1h) or the reaction of anilines with (1‐ethoxycyclopropoxy)trimethylsilane or its bromide analogue followed by reduction (Scheme 1b) [67, 68]. A significant technological advancement was achieved through modifications of the Kulinkovich [69, 70] cyclopropanols synthesis by Chaplinski and de Meijere [71] that consists in replacing esters with their amide analogs (Scheme 1c). Applying these conditions to nitrile derivatives, Szymoniak successfully synthesized unsubstituted cyclopropylamines on nitrogen with excellent yields (Scheme 1i) and broad applicability, establishing this as one of the most efficient methods for preparing these compounds [72]. Recently, C4–C3 ring contraction of α‐hydroxycyclobutanones has been proposed as a viable method for synthesizing cyclopropylamine ketones both in batch and continuous flow conditions (Scheme 1d) [3].

**SCHEME 1:**
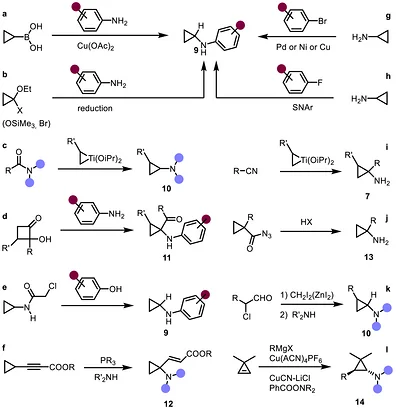
Representative synthetic strategies for the preparation of cyclopropylamine derivatives, including cross‑coupling reactions, SNAr processes, modified Kulinkovich protocols, ring‑contraction approaches, rearrangements, and recent catalytic or one‑pot methodologies.

Among historical methodologies, the Curtius amination [73, 74] (Scheme 1j) and the Smiles rearrangement [75] (Scheme 1e) are noteworthy, providing access to amino derivatives in good yields, albeit through laborious processes. In recent years, new simple and cost‐effective methods have expanded the toolkit for cyclopropylamines, such as the use of phosphines [76] to trigger amination of 3‐cyclopropylpropiolates (Scheme 1f) or one‐pot synthesis of substituted cyclopropylamines from α‐chloroaldehydes with sequential addition of CH_2_(ZnI)_2_ and the desired amine [77].

Finally, enantioselective processes [78, 79, 80] also deserve mention, particularly Marek's work, which utilizes the sequential addition of organocopper reagents and amines to cyclopropenes, and it has emerged has a valid procedure for the preparation of polysubstituted cyclopropylamine compounds (Scheme 1l) [82]. In recent years, several photoredox processes have been developed for the synthesis of cyclopropylamines. For instance, Zhang and Fang reported a cyclopropanation of *N*‐vinylimides via a redox‐neutral photocatalyzed radical addition/anionic cyclization process (Scheme 2a) [83]. Xiao and Xue, on the other hand, developed an amination reaction of aryl halides with both primary and secondary alkyl amines via Ni‐photocatalysis (Scheme 2b) [84, 85, 86].

**SCHEME 2:**
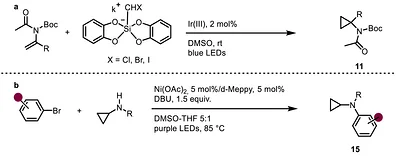
Photocatalytic strategies for the synthesis of cyclopropylamine derivatives. (a) Redox‑neutral radical addition–cyclization of *N*‐vinylimides. (b) Ni/photoredox‑mediated amination of aryl halides with primary and secondary alkyl amines.

## 2 Photoinduced Transformations of Cyclopropylamines

### 2.1 Cyclopropylimine‐1‐Pyrroline Photorearrangement

In recent decades, various studies have highlighted the reactivity of cyclopropylimine derivatives to elucidate the mechanisms associated with their photochemical transformations. These investigations have combined experimental approaches with theoretical insights, leveraging ab initio quantum mechanical calculations [87, 88].

From an experimental perspective, it has been demonstrated that the irradiation of cyclopropylimine solutions using medium‐pressure mercury lamps results in measurable quantum yields of approximately Φ = 0.002–0.005. This low quantum yield necessitates prolonged irradiation. However, these compounds exhibit notable photostability, allowing the high‐yield formation of pyrroline derivatives [89]. Interestingly, the transformation appears largely independent of the substitution pattern on the cyclopropane system. Upon photoexcitation, the compounds undergo ring‐opening followed by a 1,5‐closure, forming the corresponding photoproducts. This process has been shown to be unaffected by the nature of the solvent, ruling out the involvement of charged intermediates and instead favoring radical species [90]. As illustrated in Scheme 3, the rearrangement from cyclopropylimines to pyrrolines occurs via two stereochemically distinct pathways, which can be rationalized using the Woodward–Hoffmann descriptors [91]. Experimentally, it has been observed that *trans*‐cyclopropyl aldimines, upon irradiation, preferentially form the corresponding *trans*‐pyrrolines **17**. Similarly, *cis*‐derivatives yield *cis*‐pyrrolines **17’**, demonstrating a high degree of regioselectivity in the bond cleavage events preceding the ring expansion [92]. On the other hand, substituting the imine hydrogen atom with a bulkier functional group results in the formation of diastereomeric mixtures approaching a 1:1 ratio. This indicates that increasing steric hindrance in these systems is critical, favoring *cis*‐to‐*trans* isomerization. In this context, s and a (suprafacial, antarafacial) and r and i (retention, inversion) notation have been employed to describe the stereochemical outcomes of the 1,3‐shift process involved in this photoinduced ring‐expansion mechanism. From a purely photochemical standpoint, cyclopropyl aldimines absorb light in the 270–300 nm range, depending on the substituents present on either the imine or cyclopropane moiety. Their photoexcitation, either directly or via photosensitizers, leads to the generation of biradical intermediates. This process has been rationalized as involving the promotion of the molecule to the *S*_2_ (π–π*) excited state. Subsequent relaxation to *S*_0_ results in cyclopropane σ‐bond cleavage as the system transitions to the *S*_1_ state, associated with rotation of the imine moiety. This rotation influences the reaction's regiochemical outcomes regardless of the solvent employed. The transient biradical species then undergoes recombination, yielding the pyrroline photoproduct. The stereochemistry of this product is influenced by the stability of the radical intermediates involved in the process [93].

**SCHEME 3:**
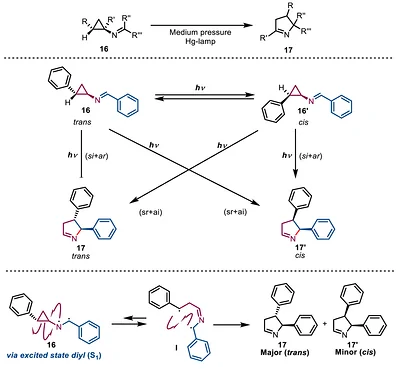
Photochemical rearrangement of cyclopropylimines to pyrrolines.

### 2.2 Photoinduced Cyclopropylamine Ring‐Opening

A novel photoredox cyclopropylamine ring‐opening nucleophilic attack mediated by Ir[dF(CF_3_)ppy]_2_(5,5’‐dCF3bpy)PF_6_ in the presence of Co(dmgH)_2_(4‐CN‐py)Cl and blue light was developed by Luan and Zuo. This catalytic process enables the synthesis of N,O‐acyl‐acetal derivatives in moderate to excellent yields through a SET process in which the acylamine is oxidized by the catalyst. Further β‐scission and iminium radical formation is followed by a dehydrogenative process mediated by the Co‐complex which generates the 1,4‐adduct with liberation of H_2_. Nucleophilic attack allows access to the desired desaturated functionalized products as reported in the Scheme 4 [94].

**SCHEME 4:**
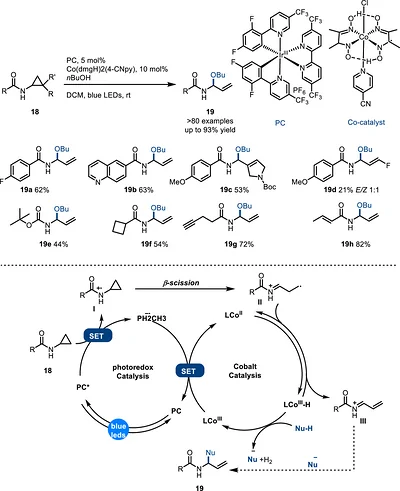
Ir‑photocatalyzed oxidative tandem cyclopropylamides ring opening‐Co‑mediated dehydrogenation and nucleophilic trapping accessing N,O‑acyl‑acetal products.

The oxidative fluorination of cyclopropylamides via organic photoredox catalysis was developed by Waser and coworkers through a two‐step, one‐pot reaction using benzophenone as a photocatalyst (PC) under UV irradiation (365 nm). Additionally, other photocatalysts, such as [Ir(dF‐CF_3_ppy)_2_(dtbbpy)]PF_6_ and Mes‐Acr^+^, could also be employed to achieve similar results under visible light irradiation. This reaction proceeds via the photoexcitation of benzophenone, which, in its excited triplet state, is reduced through electron donation by a cyclopropylamide **20**. As a result, the cyclopropylamide radical cation undergoes β‐scission, forming the distal iminium radical intermediate **II**. This intermediate reacts with a fluorinating agent, such as Selectfluor, leading to the generation of an iminium intermediate that can be attacked by a nucleophile to produce the corresponding 3‐fluoroaminal. The addition of various nucleophiles to the reaction mixture, that is methanol, thiophenols, pyrazoles, and tetrazoles, allows for the isolation of diverse N,O‐ (**21a**), N,S‐ (**21b**), or *N*,*N*‐acetals (**21c**) up to 73% yields. Furthermore, the addition of electron‐rich aromatic nucleophiles, such as indoles, pyrroles, or alkyloxybenzenes, results in the formation of the corresponding 3‐fluoroacylamines **21d–h** with good yields and broad substrate scope (Scheme 5) [95].

**SCHEME 5:**
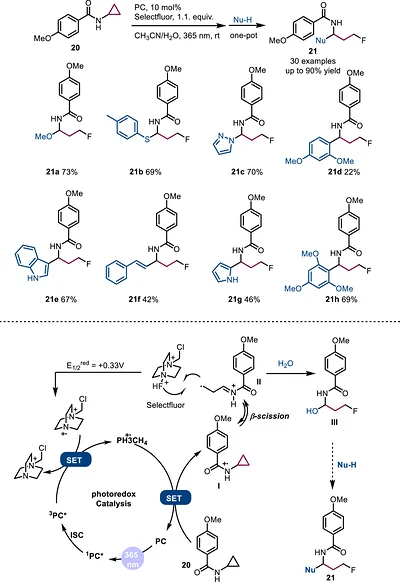
Photoinduced oxidative fluorination of cyclopropylamides using benzophenone as photocatalysts.

This photoinduced process was rationalized by the authors, considering the redox potentials of Selectfluor (*E*_1/2, red_ = + 0.79 V) [96] relative to that of the benzophenone triplet state (*E*_1/2red_ = −0.62 V) and the benzophenone radical cation (BP) which is sufficiently oxidizing to convert cyclopropylamide into its radical cation intermediate (*E*_1/2_ = +2.37 V. The radical cation generated through this single‐electron transfer (SET) process can then react with the fluorinating agent, introducing the halogen at the distal position. Simultaneously, the iminium group, reacting with water, would generate the corresponding hemiaminal. This synthetic methodology has been also applied to *N*‐arylamino cyclobutanes **22**, accessing to the desired radical cation that, prone of ring opening, generated a β‐iminium radical specie able to react with Selectfluor to install in the distal position a fluorine atom, and with a series of nucleophiles leading to the desired fluorobutyl derivatives **23** in moderate to good yields, as reported in the Scheme 6.

**SCHEME 6:**
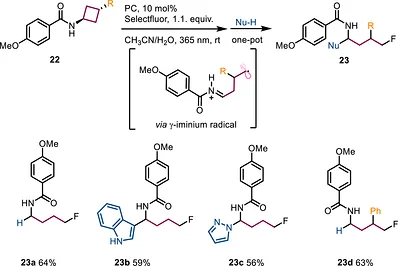
Photoinduced oxidative fluorination of cyclobutylamides using benzophenone as photocatalysts accessing fluorobutyl derivative.

Upon irradiation of *N*‐phenyl cyclopropylamine, in the presence of Ru (II) photocatalysts and air, Zheng observed the formation of an unstable endoperoxide that treated with acetylchloride can be smoothly converted into the 3‐acetoxy propionamide **24a**, as depicted in Scheme 7 [43].

**SCHEME 7:**
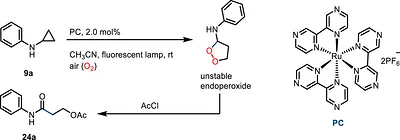
Photooxygenation of *N*‐phenyl cyclopropylamine in the presence of Ru(II) photocatalysts. Formation of unstable endoperoxide intermediates that, upon acetyl chloride treatment, afford 3‑acetoxypropionamide compounds.

### 2.3 Photoinduced Cyclopropylamine [3 + 2] Cycloadditions With Olefins

Zheng also reported a formal photoinduced [3 + 2] cycloaddition process of *N*‐aryl cyclopropylamines with terminal alkynes, catalyzed by Ru(bpz)_3_(PF_6_)_2_. Upon exposure to white light, the corresponding reaction mixtures yielded a variety of desired cyclopentenyl aniline **27** derivatives with isolated yields ranging from 20% to 91% [97]. Furthermore, replacing terminal alkynes with a series of symmetrical and asymmetrical 1,3‐conjugated diynes successfully enabled the intermolecular [3 + 2] annulation with *N*‐aryl cyclopropylaniline, leading to the formation of various conjugated cyclopentene adducts in good yields. The authors extended this synthetic process to 1,3‐conjugated enynes, which produced mixtures of isomeric products during the photocyclization process. These reactions displayed variable regioisomeric ratios but consistently showed that the [3 + 2] cycloaddition preferentially involves the interaction between the radical cation generated from the photooxidation of the cyclopropylamine and the triple bond of the alkyne, rather than the sterically more hindered olefinic portion. This preference is driven by both steric and electronic factors [98]. A selection of products resulting from this transformation is summarized in Scheme 8.

**SCHEME 8:**
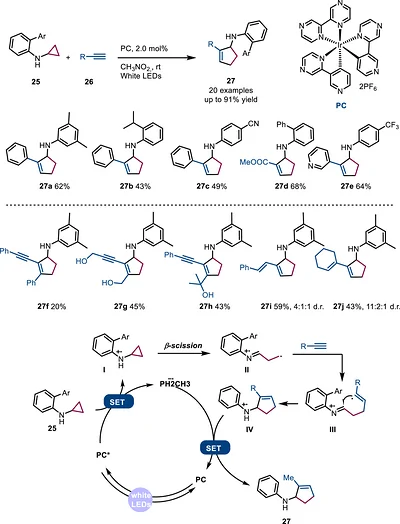
Visible‑light‑mediated [3 + 2] cycloaddition of cyclopropylamines with alkynes catalyzed by Ru(bpz)_3_(PF_6_)_2_ via SET process to furnish disubstituted cyclopentenyl aniline derivatives.

In 2021, Aggarwal and coworkers reported a novel [3 + 2] cycloaddition of *N*‐sulfonyl cyclopropylamines **28** with electron‐poor olefins to afford *trans*‐substituted amino cyclopentanes **30** with high diastereoselectivity under visible‐light irradiation, using 4CzIPN as a photocatalyst and K_3_PO_4_ as a base (Scheme 9). The choice to include a base in the reaction environment stems from the fact that sulfonamides cannot be efficiently oxidized by the triplet excited state of 4CzIPN during the photoredox process. However, the addition of a base allows the generation of the corresponding anion, which can undergo single‐electron oxidation to form an aza‐anion radical [99]. This radical subsequently undergoes ring‐opening of the cyclopropane moiety via a β‐scission mechanism. The resulting species is trapped by an electron‐deficient olefin, generating a new alkyl radical. Single‐electron reduction of this radical by the reduced state of the photocatalyst produces a stabilized carbanion, which can readily attack the *N*‐sulfonyl imine group. After protonation, this delivers the substituted cyclopentane product. This synthetic strategy was explored using various acrylate derivatives and, more broadly, employing different electron‐poor olefins. In general, this approach yielded the corresponding *trans*‐cyclopentyl sulfonimines as the major products, with good chemical yields and high levels of diastereoselectivity [100].

**SCHEME 9:**
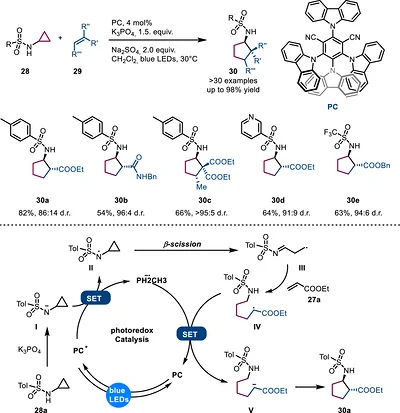
Visible‑light‑mediated [3 + 2] cycloaddition of *N*‐sulfonyl cyclopropylamines with electron‑deficient olefins using 4CzIPN and K_3_PO_4_, affording *trans*‐substituted cyclopentanes via anion‑radical‑initiated β‑scission and radical cyclization sequence.

In the same year, Gianetti's group reported a photoinduced [3 + 2] cycloaddition of cyclopropylamine with olefins, catalyzed by DMQA^+^ under red light (640 nm) irradiation, leading to the synthesis of cyclopentylamines **32** in good yields but with low diastereoselectivity [101, 102, 103]. Using the same reaction conditions, the [3 + 2] cycloaddition of cyclopropylamine with both electron‐deficient and electron‐rich terminal alkynes **31** was successfully achieved, providing access to a broad range of cyclopentene substrates in 70%–80% yield (Scheme 10). The authors have proposed a mechanism to rationalize this transformation. First, photoexcitation of nPr‐DMQA with red light initiates a SET process, oxidizing cyclopropylamine and generating the *N*‐aryl nitrogen radical cation intermediate (Intermediate **I**). Subsequently, β‐scission of this intermediate produces a distonic iminium radical, which reacts with the alkene to form a new radical cation species (Intermediate **III**). Intramolecular radical cyclization of Intermediate **III** then yields the cyclopentylamine framework. Finally, reduction of this intermediate by nPr‐DMQA regenerates the active catalyst and provides the desired cyclopentene products.

**SCHEME 10:**
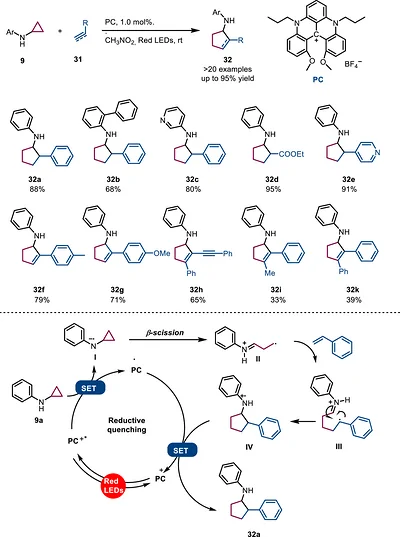
Red‑light‑mediated [3 + 2] cycloaddition of *N*‐aryl cyclopropylamines with alkynes catalyzed by DMQA^+^, to afford cyclopentylamine or cyclopentene products.

Very recently, Barriault and coworkers observed that the [3 + 2] cycloaddition of *N*‐aryl cyclopropylamines and acrylates could be achieved through a catalyst‐free, photochemical process [104]. During their studies, the authors noted that in background reactions conducted in the absence of a photocatalyst, under both 365 and 465 nm irradiation, the corresponding cyclopentylamine was formed in high yields after 24–48 h of irradiation of a solution of the two reagents in acetonitrile (Scheme 11). Following this procedure, numerous aminocyclopentyl derivatives **33** were obtained, with chemical yields ranging from 57% to 99% and diastereomeric ratios (*d.r*.) between 1:1 and 9:1, depending on the degree of substitution and electronic properties of the employed olefins. To rationalize this result, the authors conducted UV–vis measurements, which revealed the probable formation of an electron donor‐acceptor (EDA) complex between the cyclopropylamine (donor) and ethyl acrylate (acceptor). Upon absorption of light by the complex, a SET is triggered, oxidizing the donor to generate the corresponding radical cation, followed by β‐scission to produce the usual iminium radical cation. Simultaneously, the acceptor undergoes reduction, generating a β‐radical anion. These radical species recombine, leading to the formation of the desired product.

**SCHEME 11:**
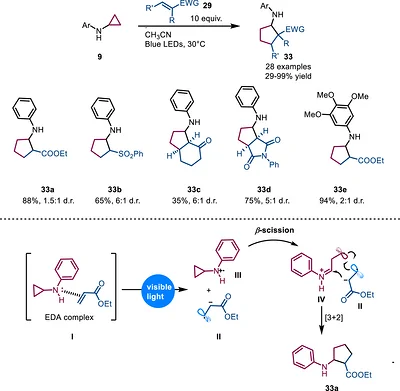
Catalyst‐free [3 + 2] photocycloaddition of *N*‐aryl cyclopropylamines with acrylates via an EDA complex.

Waser and collaborators reported a visible‐light‐mediated [3 + 2] cycloaddition of cyclopropylamines and cyclopropene derivatives **34** as a convergent and highly diastereoselective strategy for the synthesis of bicyclo[3.1.0]hexanes **35** containing an all‐carbon quaternary center (Scheme 12). This reaction is facilitated by photoredox catalysis using 4CzIPN and blue LED irradiation, allowing access to the desired compounds with three consecutive stereocenters in good yields and a broad substrate scope. The introduction of easily removable functional groups on the amino residue further enabled access to various important building blocks for medicinal chemistry [44].

**SCHEME 12:**
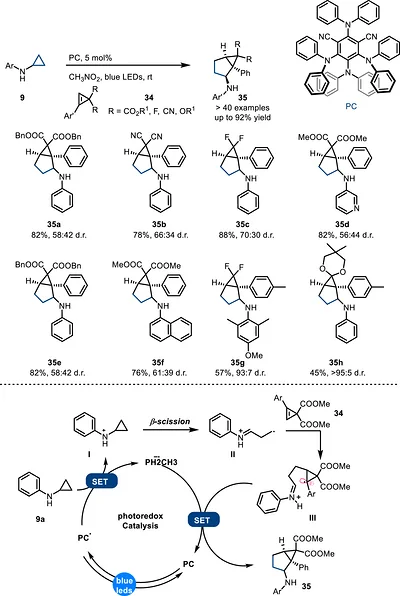
Diastereoselective synthesis of bicyclo [3.1.0] hexanes bearing an all‐carbon quaternary center and three contiguous stereocenters via photoredox [3 + 2] cycloaddition of cyclopropylamines and cyclopropenes.

Albrecht and coworkers developed an innovative strategy for the synthesis of cyclopenta[b]chromeno‐carbonitriles **37** by reacting 3‐cyanochromones **36** with *N*‐cyclopropylamines **9** in DMSO under green light irradiation (525 nm), using Eosin Y as the photocatalyst. Under these operational conditions, Eosin Y in its excited triplet state facilitates the oxidation of the cyclopropylamine derivative via a SET process. This oxidation promotes the ring‐opening of the cyclopropane moiety, leading to the formation of the distonic radical species **II**. This radical species subsequently attacks the chromone derivative, generating a secondary radical. The latter undergoes reaction with the iminic group, efficiently closing the cyclopentane system. The final step involves electron transfer from the reduced photocatalyst to the *N*‐aryl moiety, enabling the formation of the desired products as mixtures of diastereoisomers with chemical yields ranging from 58% to 80%, while simultaneously regenerating the photocatalyst. This process is summarized schematically in Scheme 13 [105].

**SCHEME 13:**
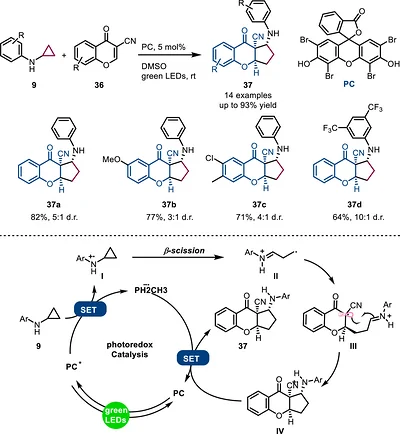
Eosin‑Y‑catalyzed green‑light [3 + 2] cycloaddition of 3‑cyanochromones with *N*‐cyclopropylamines. Synthesis of cyclopenta[b]chromeno‑carbonitriles.

An efficient radical cascade reaction to synthesize 3‐aminotetrahydro‐1H‐carbazoles **39** (Scheme 14) was developed by Zhang, Zhu, and Yao [106]. The method involves reacting 2‐alkenylarylisocyanides **38** with cyclopropylanilines **9** under visible‐light irradiation at room temperature, using Ir(dF(CF_3_)ppy)_2_(dtbbpy)PF_6_ as a photocatalyst. This synthetic methodology demonstrates a broad substrate scope and provides the corresponding carbolines in good to excellent chemical yields and diastereoselectivities ranging from 1.3:1 to >20:1, depending on the substituents present on the aryl portions of the cyclopropylamines or isocyanides. The proposed mechanism to rationalize this transformation involves the formation of an iminium radical cation via β‐scission of the cyclopropylamines through a reductive quenching of the Ir(III) photocatalyst. The newly generated iminium radical cation is trapped by the 2‐alkenylarylisocyanide to produce radical intermediate **III**. This species undergoes a 5‐*exo*‐*trig* cyclization, followed by intramolecular addition to the iminium ion, forming nitrogen radical cation **V**. This cation is subsequently reduced by the photocatalyst, completing the catalytic cycle. The final step involves tautomerization from the pyrroline form to the indole‐derived structure, yielding the desired 3‐aminotetrahydro‐1*H*‐carbazole products.

**SCHEME 14:**
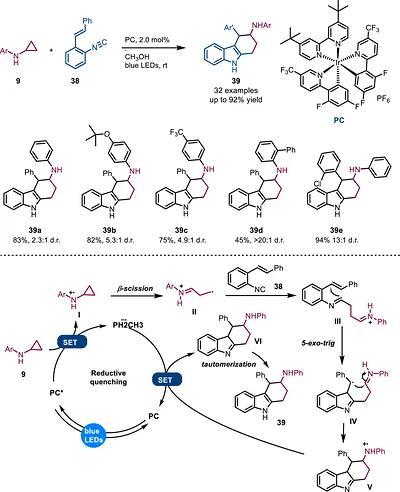
Visible‑light‑mediated radical cascade between 2‑alkenylarylisocyanides and cyclopropylanilines using Ir(dF(CF_3_)ppy)_2_(dtbbpy)PF_6_. Sequential radical cyclizations to deliver 3‑aminotetrahydro‑1*H*‑carbazoles.

The synthesis of arylamino cyclohexenes **41** via photocatalysis, based on the reaction between *N*‐aryl cyclopropylamines **9** and vinyldiazo compounds **40** using 4CzIPN as a photocatalyst, was reported by Zhou in 2024 (Scheme 15). Following the generation of the iminium radical cation through the reductive quenching of the catalyst in its excited state, this species is attacked by the nucleophile diazo derivative. This sequence first forms a C─C bond, followed by the attack of the distal radical on the vinyl portion. Finally, single‐electron reduction of this intermediate by the photocatalyst (PC*) and the subsequent loss of nitrogen lead to the formation of the desired 2‐cyclohexene amines [107].

**SCHEME 15:**
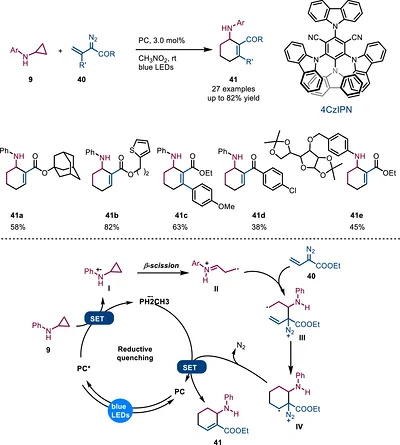
4CzIPN‐catalyzed [3 + 2]‐type annulation of cyclopropylamines with vinyldiazo compounds to access arylamino cyclohexenes.

An original example of cycloexpansion of cyclopropylamine derivatives was recently reported by Secci and coworkers, employing 4CzIPN as a photocatalyst to access 2,2‐ and 2,6‐functionalized pyrrolines **43** in good to excellent chemical yield and broad substrate scope [108]. In this study, cyclopropyl enamines are converted into the radical cation **I** via an oxidative SET process. Subsequent electronic reorganization and ring opening generate intermediate **II**, which undergoes intramolecular radical addition to the *N*‐acryl enamine moiety, affording the pyrroline radical cation **III**.

Finally, reductive quenching via SET from the photocatalyst (PC^.−^) furnishes the desired products **43** and regenerates the catalyst, as depicted in Scheme 16.

**SCHEME 16:**
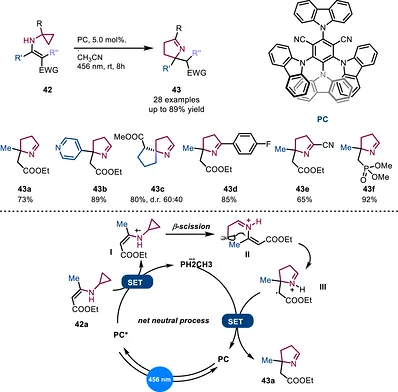
4CzIPN‑mediated ring opening and radical cyclization of *N*‐cyclopropylenamines delivering functionalized pyrrolines via SET process.

Zheng and collaborators reported an elegant synthetic protocol to obtain aminocyclopentyl systems‐derived from photoredox reactions initiated by the oxidation of cyclopropylamines, followed by their subsequent reaction with olefins. This approach employs diastereomerically pure [3.1.0] bicyclic derivatives, which are reacted with styrene to produce the desired products [43]. These compounds efficiently generate the corresponding radical cations through an oxidative quenching process involving [Ru(bpz)_3_](PF_6_)_2_·2H_2_O in CH_3_NO_2_ under aerobic conditions. Under these reaction conditions, the authors achieved a range of bicyclic derivatives with good to excellent chemical yields and *cis*/*trans* diastereomeric ratios varying from 4:1 to 25:1, depending on the functional groups introduced into the starting [3.1.0] bicyclic derivatives. Additionally, [4.1.0] bicyclic cyclopropylamine participated in the cycloaddition similarly, yielding 6,5‐fused bicyclic heterocycles **45** with a predominant *cis* geometry (4:1 dr) and a yield of 58% (Scheme 17). These results were rationalized through a reaction mechanism involving electron transfer from the cyclopropylamine to the Ru^2^^+^ complex upon visible‐light photoexcitation. The nitrogen radical cation subsequently undergoes ring cleavage to generate a β‐carbon radical‐iminium ion intermediate **II**, which is trapped by the olefin. The intramolecular addition of the stabilized radical **III** to the iminium ion occurs via a Beckwith–Houk chair‐like transition state, which minimizes steric interactions between R and Ph [109]. This process forms the nitrogen radical cation **IV**, which leads to the construction of the bicyclic system. The intermediate is then reduced by Ru^1^^+^, completing the catalytic cycle.

**SCHEME 17:**
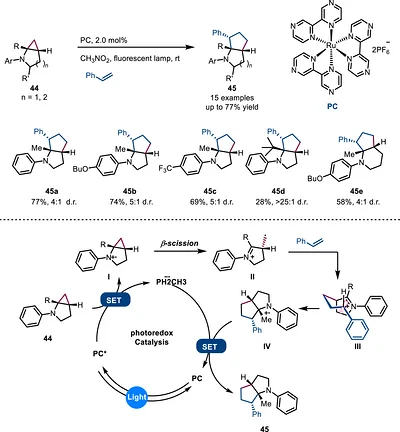
Photoredox‐catalyzed [3 + 2] cycloaddition of [3.1.0] bicyclic derivatives with olefins to access bicyclic pyrrolidin and pyperidin products.

Recently, a powerful method to access to *gem*‐difluorinated cyclopentylamines via photocatalytic [3 + 2] cyclopropylamines ring opening‐intramolecular cyclization has been proposed by Qiu and coworkers [110]. Upon blue LEDs irradiation, the excited Ir‐based photocatalyst reacts with the cyclopropylamine 9 generating the radical cation I that, after ring‐opening, leads to radical specie II. Radical addition of II to the *gem*‐difluorinated alkene 46 leads to the formation of the intermediate III that undergoes intramolecular cyclization to IV. In the end, IV is reduced by the photocatalyst in its radical anion form releasing the product 47 as reported in the Scheme 18.

**SCHEME 18:**
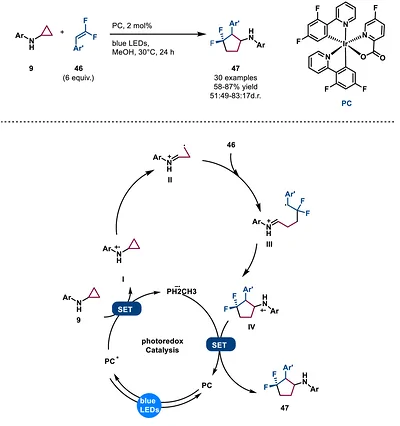
Photoinduced synthesis of *gem*‐diflurinated *N*‐aryl cyclopentylamines via [3 + 2] cycloaddition of *N*‐aryl cyclopropylamine with fluorinated olefins.

In a similar way, the photochemical, tricomponent [3 + 2] cyclization reaction featuring cyclopropylamines as starting materials and water as solvent has been proposed by Xiao and An [111]. Their approach features the in situ Knoevenagel reaction between an aldehyde and an activated methylene and the contemporary photochemical activation of the cyclopropyl amine 9, leading to the formation of cyclopentylamines bearing two geminal electron‐withdrawing group 50 (Scheme 19).

**SCHEME 19:**
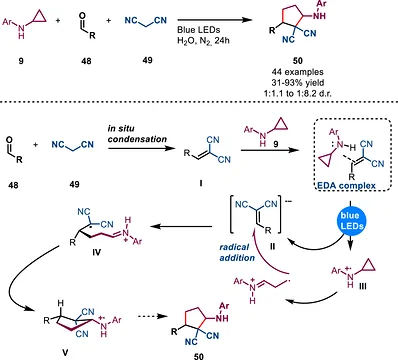
In situ photochemical tricomponent [3 + 2] cyclization of *N*‐aryl cyclopropylamine with 2‐substituted cyan nitriles to access 2,2‐dicyano‐*N*‐aril cyclopentylamine via EDA‐complex.

Another example of [3 + 2] photocatalyzed cyclization has been reported by Tang and coworkers: here the 9‐mesityl‐10‐methyl acridinium perchlorate has been employed to activate the cyclopropylamide **51**, enabling it to react with *N*‐substituted maleimides **52** to form bicyclic *N*‐acyl cyclopentylamine derivatives **53** under mild reaction conditions and without the use of additives (Scheme 20) [112].

**SCHEME 20:**
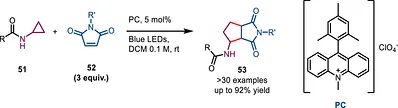
Acridinium salt mediated photocatalytic [3 + 2] cycloaddition of *N*‐acyl cyclopropylamides with *N*‐substituted maleimides to afford arylamino tetrahydrocyclopenta[c]pyrrole diones.

### 2.4 Photoinduced Enantioselective Cyclopropylamine [3 + 2] Cycloadditions With Olefins

An enantioselective [3 + 2] cycloaddition between *N*‐aryl cyclopropylamines and α‐branched 2‐vinylazaarenes **54** was reported by Huang and Jiang in 2020 [113]. This synthetic methodology relies on a cooperative photoredox and chiral Brønsted acid catalysis, involving a sequential two‐step process: (a) SET oxidation of the cyclopropylamine derivatives to generate radical cations, followed by a ring‐opening sequence to produce the distonic iminium radical cation; (b) trapping of the intermediate with an alkene, leading to a radical addition to the iminium ion. This process enables access to a broad range of 2,2‐disubstituted *N*‐aryl cyclopentylamines **55**, achieving good to excellent yields, high enantioselectivity (up to 96% *ee*), and diastereoselectivity (d.r. >19:1). To unlock this transformation, 9,10‐dicyanoanthracene (DPZ) was employed as the photocatalyst under blue‐light irradiation and the enantioselectivity was ensured by the use of imidodiphosphoric acids. Through a combination of photophysical studies, experimental investigations, and quantum mechanical calculations, the authors elucidated that the transformation is triggered by the reductive quenching of DPZ by cyclopropylamines generating the iminium radical species likely interacts with the imidodiphosphoric acid via the formation of a hydrogen‐bonding network (TsA^+^), facilitating the generation of two new stereocenters with a high degree of stereochemical control (Scheme 21).

**SCHEME 21:**
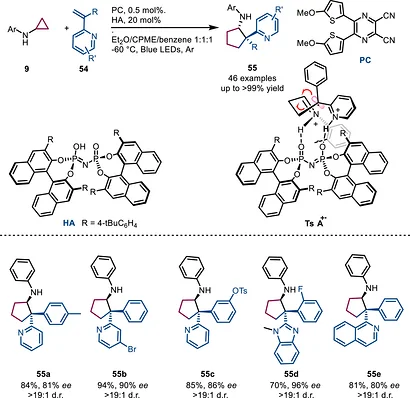
Enantioselective [3 + 2] cycloaddition of *N*‐aryl cyclopropylamine with 2‐vinylpyridines catalyzed by DPZ in the presence of imidodiphosphoric acids to access to enantiomerically enriched cyclopentylamines.

In the same year, Ooi and collaborators developed an innovative synthetic approach for the preparation of enantiomerically enriched cyclopentylamines (Scheme 22) via a [3 + 2] cycloaddition of *N*‐cyclopropylurea derivatives **56** with the alkenes **57** [114]. This approach relies on the generation of a reactive radical intermediate within a supramolecular assembly formed by non‐covalent interactions between a borate chiral catalyst and an *N*‐cyclopropylurea derivatives. This system also anchors a racemic photocatalyst, [Ir(dFCF_3_ppy)_2_(dtbbpy)]^+^, forming a supramolecular ion pair. Upon light irradiation, the excited cationic Ir complex oxidizes the ureido derivative, generating the corresponding radical‐ion pair (**II**). This intermediate undergoes β‐scission within a defined three‐dimensional arrangement (**IV**). The resulting radical cation reacts with the alkenes in a stereoselective manner within the asymmetric environment created by the chiral anion (**V**). Further stereodetermining *five‐exo* cyclization step, has been evoked to form the aminocyclopentylurea radical cation (**VI**). Then, oxidative quenching of the photocatalyst delivers the desired aminocyclopentylurea compounds **58** with high enantioselectivity.

**SCHEME 22:**
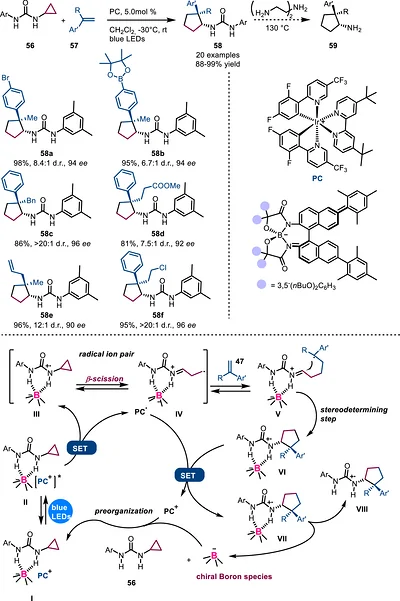
Enantioselective [3 + 2] cycloaddition of *N*‐cyclopropylureas with alkenes via supramolecular ion‑pair photoredox catalysis. SET oxidation within a chiral borate–urea assembly induces β‑scission and radical addition–cyclization, affording enantioenriched aminocyclopentylureas.

Zhao, Cao, and Jiang reported a series of studies on enantioselective [3 + 2] photoaddition processes involving distonic radical intermediates generated via ring opening of *N*‐aryl cyclopropylamines **9**, enabled by SPINOL‐based chiral phosphoric acids [45]. The stereochemical outcome is governed by a hydrogen‐bonding interaction between the Brønsted acid catalyst (HA) and the amine substrate, forming a preorganized complex that undergoes single‐electron oxidation by the photoexcited DPZ catalyst. This step triggers cyclopropane ring opening to generate the corresponding distonic iminium radical. Subsequent hydrogen‐bonding interactions with the chiral phosphoric acid guide the addition to electron‐deficient 3‐methylene‐isoindolin‐1‐ones **60**, furnishing a tertiary benzyl radical intermediate. Intramolecular cyclization then delivers the corresponding spirocyclic products **61** with high levels of enantio‐ and diastereocontrol (Scheme 23a).

**SCHEME 23:**
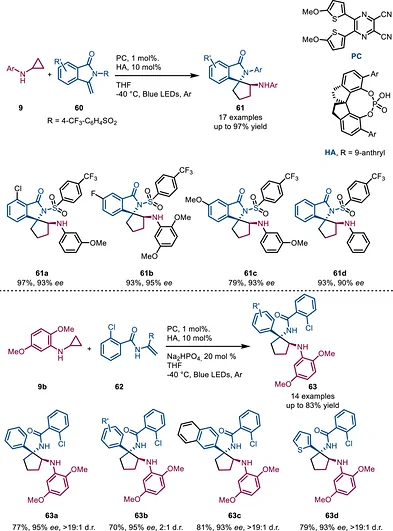
Photoinduced enantioselective [3 + 2] cycloaddition of *N*‐aryl cyclopropylamines with 3‑methylene‑isoindolin‑1‑ones promoted by SPINOL chiral phosphoric acids and DPZ to furnish (a) spirocyclic products and (b) enantiomerically enriched *N*‐aryl cyclopentylamines.

The protocol provides a broad range of spiro compounds in good‐to‐excellent yields and diastereomeric ratios exceeding 19:1, depending on the electronic and steric properties of the substrates. These findings are consistent with earlier reports by Zheng, who demonstrated the reactivity of radical cations generated via β‐scission of cyclopropylamines in [3 + 2] cycloadditions with electron‐rich olefins [45]. Notably, this strategy was further extended to reactions with *N*‐benzoyl enamines (Scheme 23b), enabling direct access to enantiomerically enriched *trans*‐diaminocyclopentyl derivatives **63** with excellent diastereoselectivity (d.r. >19:1).

Building on these results, the authors further explored the reactions of *N*‐cyclopropyl arylamines with electron‐neutral diaryl olefins **64** to extend the applicability of this remote hydrogen‐bonding induction strategy. Successfully, the use of olefins bearing OTf groups resulted in the formation of the corresponding α,α‐diaryl cyclopentylamines **65** with excellent yields and high levels of enantio‐ and diastereoselectivity. A plausible reaction mechanism for these transformations is proposed in the Scheme 24.

**SCHEME 24:**
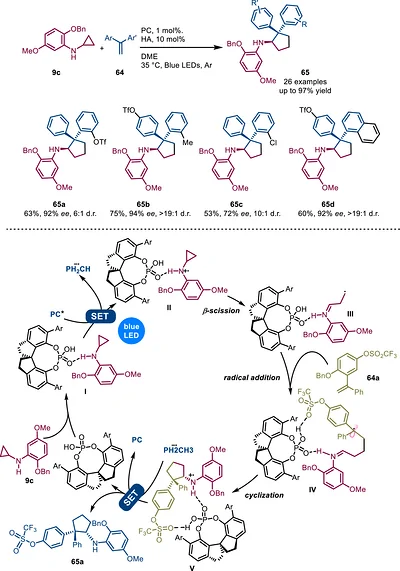
Enantioselective [3 + 2] cycloaddition of *N*‐aryl cyclopropylamines with OTf‐substituted diaryl olefins catalyzed by SPINOL chiral phosphoric acids.

Moreover, in a further application of this synthetic strategy, the same research group extended it to C6 and C7 benzofused enones **66** and **68**, leading to the formation of the corresponding spiro derivatives **67** and **69** with moderate chemical yields and good‐to‐excellent diastereoselectivity, as shown in Scheme 25 [115].

**SCHEME 25:**
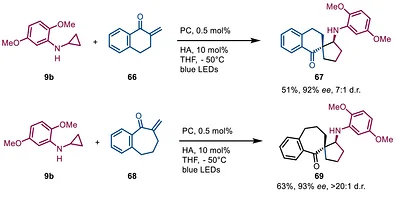
Photoinduced enantioselective [3 + 2] cycloaddition of *N*‐aryl cyclopropylamines with C6‑ and C7‑benzofused enones mediated by SPINOL chiral phosphoric acids and DPZ as a photocatalyst.

Morken and collaborators reported a stoichiometric enantio‐ and diastereo‐selective [3 + 2] photocyclization of *N*‐aryl cyclopropylamines **2** with alkenylB(sam)‐derived auxiliaries, catalyzed by Ir[dF(CF_3_)ppy]_2_(dtbbpy)PF_6_ and providing the corresponding boronyl derivatives (**I**) that after oxidative work‐up with hydrogen peroxide/NaOH, gave direct access to *trans*‐substituted aminocyclopentanols **71**, in moderate‐to‐good chemical yields and high enantiomeric excesses (Scheme 26) [116].

**SCHEME 26:**
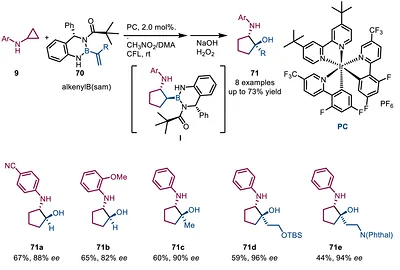
Enantio‑ and diastereo‐selective [3 + 2] photocyclization of *N*‐aryl cyclopropylamines with alkenylB(SAM) auxiliaries using Ir[dF(CF_3_)ppy]_2_(dtbbpy)PF_6_ as a photocatalyst.

Another asymmetric strategy involving the capture of iminium radical cations by imidazol‐2‐ylbut‐2‐enones 72 under photoredox catalysis has been developed by Fraile and Aleman [117]. In this protocol, the chiral ΔRh(*S*) photocatalyst is able to coordinate the α,β‐unsaturated ketone that under irradiation with visible light reacts with the distonic radical cation to perform a Giese‐type addition‐ring closure reaction accessing to tri‐substituted cyclopentyl amine derivatives 73 in moderate yields and up to 93% ee (Scheme 27).

**SCHEME 27:**
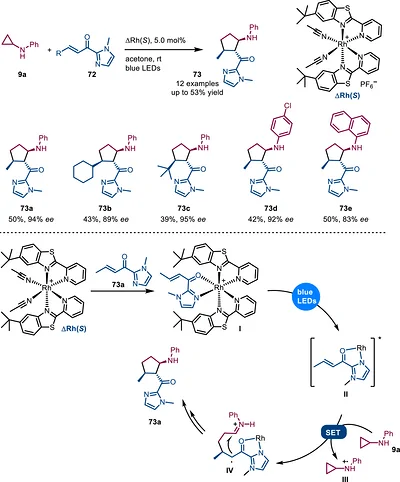
Enantioselective formation of tri‐substituted cyclopentyl amines via Δ‑Rh(*S*) photocatalysis.

Recently, another example of oxidative scission of cyclopropylamines followed by radical cations addition to enones and enynes in presence of binuclear Rh‐based catalysts that do not proceed via photoinduced oxidative quenching has been reported [118]. In this protocol, the formation of a neutral radical on the nitrogen atom and its coordination to the organometal complex has been supposed. This adduct would undergo a β‐scission and addition to the unsaturated triple bond of the acceptor, leading to the formation of substituted cyclopentylamines [101, 102, 103].

### 2.5 Photoinduced Cyclopropylamines as Valuable Starting Materials for the Synthesis of Unconventional Building Blocks

Stephenson and coworkers investigated masked cyclopropylimines **74** as viable substrates for the development of a novel multi‐step photoinduced radical fragmentation/cyclization sequence to synthesize 1‐imino norbornyl derivatives **75** [119]. The reaction takes place when a solution of cyclopropyl *p*‐nitrophenyl imines is irradiated at 390 nm in acetonitrile. This methodology offers an effective alternative to the traditional preparation of such molecular scaffolds, bypassing classic Hofmann or Curtius rearrangements of terpene‐based materials [120] or the downstream products of cyclopentadiene‐acrylate Diels–Alder reactions [121, 122, 123]. The reaction mechanism begins with the photoexcitation of the Schiff base to its singlet excited state (n, π*). This triggers the homolytic cleavage of the carbon‐nitrogen double bond [124]. The strain‐driven opening of the cyclopropane ring follows, producing an open‐shell aliphatic radical that (see Scheme 28) in the presence of an olefin residue, undergoes a 6‐*exo‐trig*‐cyclization. The newly formed radical then reacts with the imine moiety, leading to a *5‐exo‐trig*‐cyclization that constructs the characteristic bicyclic norbornyl framework. The termination of the process has been rationalized by the authors as a recombination of adjacent radicals, regenerating the Schiff base and yielding the desired products in good chemical yields and selectivity.

**SCHEME 28:**
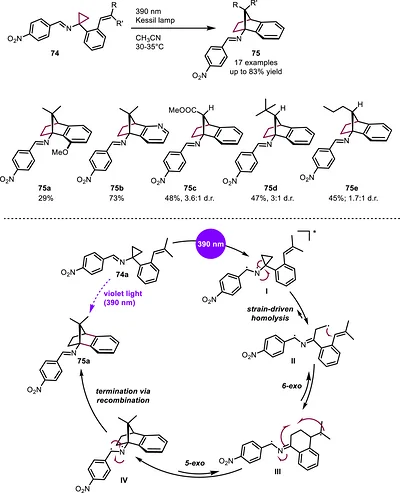
Photoinduced radical fragmentation‐cyclization of cyclopropyl *p*‐nitrophenyl imines. Excitation of the Schiff base triggers C═N homolysis and cyclopropane ring opening, followed by 6‑*exo‑trig* and 5‑*exo‑trig* cyclizations to furnish 1‑imino‑norbornyl derivatives.

Bicyclo[1.1.0]butanes **76** have garnered increasing attention in recent years due to their reactivity with various reagents, leading to ring‐opening reactions [125, 126, 127]. Aggarwal and collaborators have developed an unprecedented synthetic methodology in which bicyclo[1.1.0]butanes react with *N*‐aryl cyclopropylamines **9** via a radical mechanism. This approach provides uniquely functionalized bicyclo[3.1.1]heptanes **77** with diverse substituents, which have recently been studied for their three‐dimensional structure and potential as bioisosteres of *meta*‐substituted arenes [128, 129]. The authors describe the mechanism leading to these compounds, which involves the formation of a radical cation via β‐scission of the cyclopropylamine upon its oxidation through a reductive quenching process with the Ir(III) photocatalyst. The resulting distonic radical intermediate adds to bicyclo[1.1.0]butanes, forming a more stabilized distonic secondary benzylic radical cation (**III**). This intermediate undergoes cyclization to generate radical cation **IV**. At this stage, the newly generated adduct can be reduced either by the Ir(II) species or by another molecule of cyclopropylamine to furnish the desired compound **77** (Scheme 29). Targeted studies to elucidate this reaction mechanism have highlighted that this proposed mechanism does not fully account for a closed catalytic cycle. Instead, a propagative mechanistic pathway has been hypothesized, similar to those observed in previous photoinduced reactions involving cyclopropylamines [130].

**SCHEME 29:**
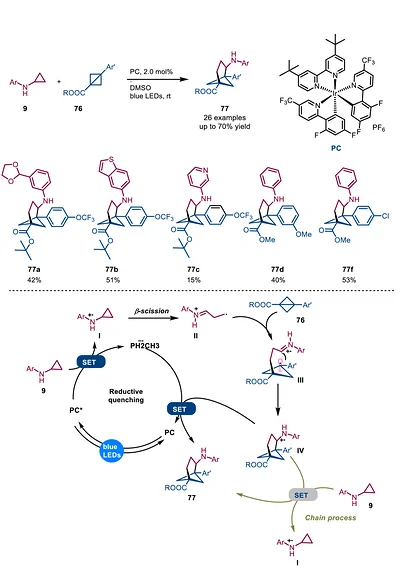
Radical addition of cyclopropylamine‐derived distonic radicals to bicyclo[1.1.0]butanes.

Once discussed the major achievements in the use of cyclopropylamines as a valuable source of β‐iminium radical species under photocatalytic conditions, we need to highlight the main features of this reactivity. A key element governing the efficiency of photoinduced cyclopropylamine activation is the thermodynamic match between the oxidation potential of the amine substrate and the excited‐state redox properties of the photocatalyst [131]. Cyclopropylamines typically exhibit high oxidation potentials, reflecting both the electron‐rich character of the amine nitrogen and the stabilizing influence of the adjacent strained ring. Reported values for *N*‐aryl cyclopropylamines generally fall in the range from +0.89 to +1.3 V versus SCE, while *N*‐alkyl derivatives tend to show slightly lower potentials due to reduced aromatic stabilization of the radical cation [44, 94, 95, 96, 97, 98, 99, 100, 101, 102, 103, 104, 105]. In contrast, the photocatalysts most frequently employed in these transformations span a broad redox window. Strong photooxidants such as Mes‐Acr^+^, 4CzIPN, DPZ, and selected Ir(III) complexes possess excited‐state oxidation potentials sufficiently positive to trigger single‐electron oxidation of cyclopropylamines, with values ranging from +0.79 to +1.27 V versus SCE. Meanwhile, milder oxidants such as Ru(bpz)_3_^2^^+^ or Eosin Y remain effective for specific substrate classes when assisted by electron‐donating substituents, base‐promoted deprotonation, or EDA‐complex formation. To guide the reader through these relationships, Scheme 30 summarizes representative oxidation potentials of commonly used cyclopropylamines in comparison with the excited‐state redox potentials of widely employed photocatalysts. This visual overview highlights the thermodynamic compatibility between substrate and catalyst classes and illustrates how subtle modifications in substitution patterns or catalyst selection can dramatically influence the feasibility of SET activation. Such redox considerations play a central role in determining whether a given transformation proceeds via oxidative quenching, reductive quenching, or EDA‐mediated electron transfer, and therefore dictate the mechanistic pathway available for β‑scission and subsequent radical reactivity [39, 132].

**SCHEME 30:**
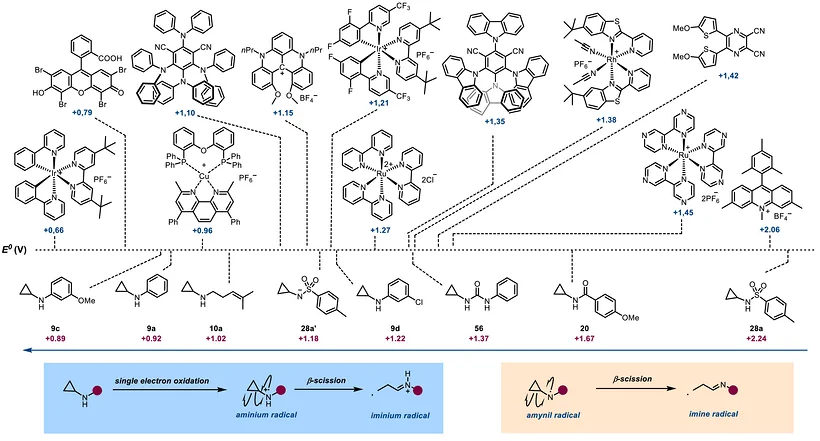
Redox potentials of cyclopropylamines plotted against the excited‑state oxidation potentials of widely used photocatalysts. The diagram illustrates the thermodynamic window required for efficient SET oxidation, enabling β‑scission and formation of the corresponding distonic iminium radicals.

## 3 Summary and Outlook

The reactivity of cyclopropylamines under photoinduced single‐electron oxidation has undergone a remarkable renaissance, transforming these strained substrates into versatile radical precursors for modern synthetic chemistry. The unique ability of cyclopropylamines to generate distonic iminium radicals with precise spatial and electronic characteristics has enabled a wide repertoire of ring‐opening functionalization, formal [3 + 2] cycloadditions, and complex radical cascades under exceptionally mild conditions. As demonstrated across the studies reviewed herein, the merger of photocatalysis, strain‐release dynamics, and intelligent substrate design has produced synthetically powerful yet mechanistically elegant transformations with broad implications for molecular synthesis.

Despite impressive progress, several challenges define the next stage of development. The reliance on high‐energy oxidants or precious‐metal photocatalysts in some protocols underscores the need for more sustainable activation modes, particularly organic dyes, EDA complexes, and metal‐free redox platforms capable of accessing the same high‐energy radical cation manifolds. Furthermore, expanding the synthetic space beyond the predominant cyclopentane frameworks toward remote functionalization, polycyclic architectures, and integrated multi‐component processes represents an exciting direction. Here, achieving fine control over radical lifetimes, spin distributions, and PC‐substrate interactions will be key.

Mechanistic insight continues to play a decisive role. Emerging spectroscopic, computational tools, artificial intelligence‐driven synthesis and automation, will improve our understanding of the short‐lived distonic intermediates governing selectivity and reactivity [133, 134, 135, 136, 137, 138, 139, 140, 141]. This deeper mechanistic resolution will likely reveal previously inaccessible radical pathways and enable new strategies for enantioinduction, diastereocontrol, and functional group economy. The chemistry of cyclopropylamines is particularly well‐positioned for such advances due to the inherent programmability of the β‐scission event and the predictable evolution of the resulting radical cation structures.

From an applied perspective, cyclopropylamine‐based photochemistry aligns strongly with current demands in drug discovery [142], agrochemical innovation, and complex molecule synthesis, where compact, three‐dimensional scaffolds and stereochemical precision are increasingly valued. Many of the transformations highlighted herein provide step‐efficient access to densely functionalized, medicinally relevant motifs, offering significant advantages over traditional multi‐step strategies. As photochemical technologies continue to merge with flow chemistry, catalyst recycling, and late‐stage functionalization, the translation of these methodologies into industrial settings appears increasingly feasible [3, 143, 144, 145].

Overall, photoinduced cyclopropylamine chemistry has transitioned from a niche concept to a powerful synthetic paradigm. Given the expanding toolbox of catalysts, activation modes, and mechanistic strategies, further breakthroughs, particularly in asymmetric catalysis, catalytic efficiency, and molecular diversity are anticipated. The field is now well‐positioned to deliver not only new reactivity but also transformative strategies for complex molecule construction across academic and industrial laboratories.

## Conflicts of Interest

The authors declare no conflicts of interest.

## Data Availability

The data that support the findings of this study are available from the corresponding author upon reasonable request.
